# Supplementary material for: Stress-specific NONO interactomes reveal a key role of Hsp70 chaperone activity in regulation of paraspeckle formation
Source: J Cell Sci. 2026 Jan 23;139(2):jcs264115. doi: 10.1242/jcs.264115 (PMC12863303; doi:10.1242/jcs.264115)
Supplement: Supplementary information [file joces-139-264115-s1.pdf]

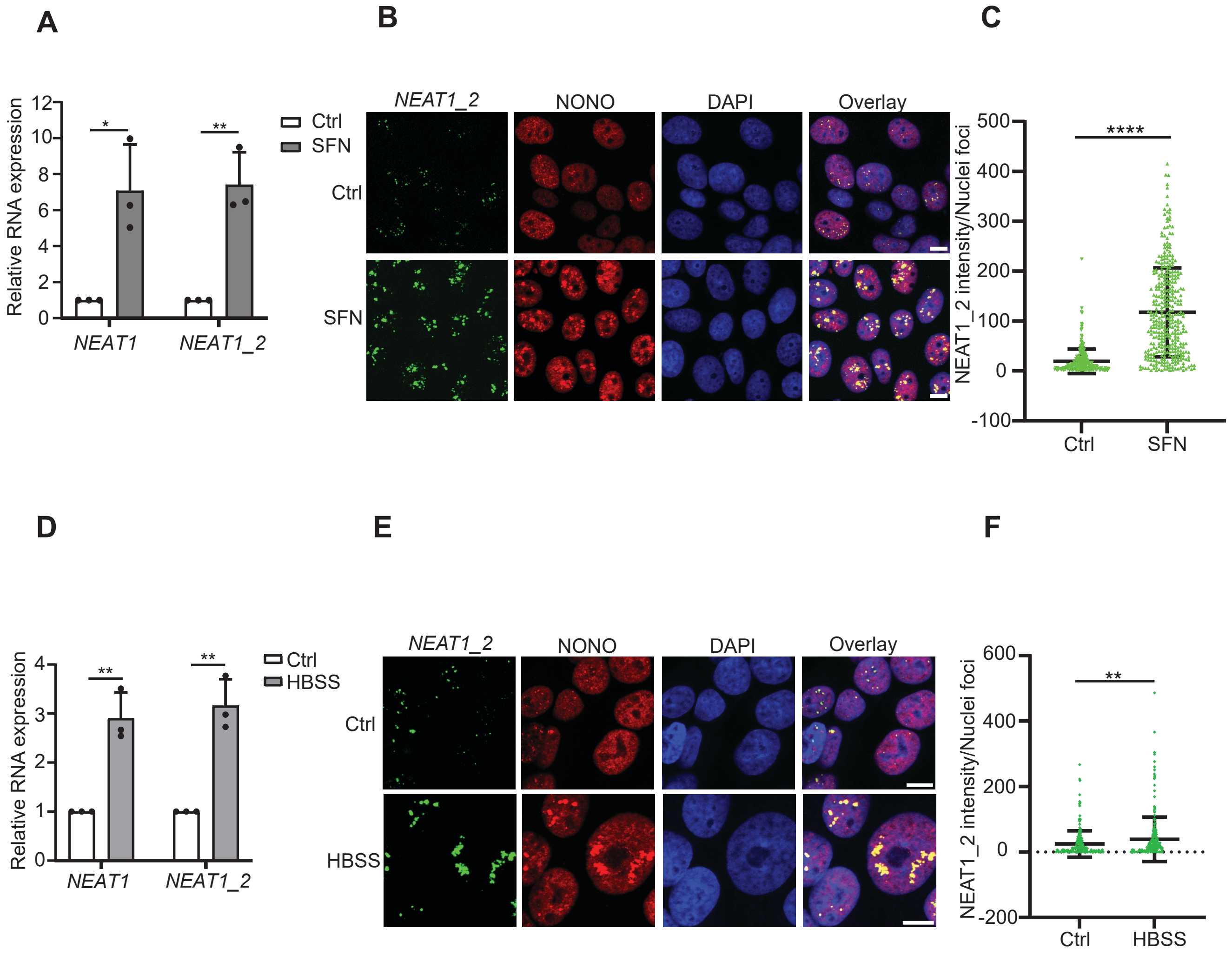

Journal of Cell Science • Supplementary information

Fig. S1. *NEAT1* expression and paraspeckle formation are increased in MCF7 cells in response sulforaphane and starvation. (A) MCF7 cells were left untreated or treated with sulforaphane (SFN) for 6 h and the relative expression of *NEAT1* (*NEAT1_1* + *NEAT1_2*) and *NEAT1_2* in treated versus untreated cells was determined by RT-qPCR. (B) Cells were treated as described in (A) and subjected to *NEAT1_2*- (green) and NONO- (red) specific co-immuno-FISH analyses. The nuclei are visualized by DAPI (blue). Scale bar is 10µm. (C) *NEAT1_2* signals from the experiment described in (B) were quantified in at least 200 cells per condition from three independent experiments. (D) MCF7 cells were cultivated in full media or in Hanks' Balanced Salt Solution (HBSS) for 6 h, and the relative expression of *NEAT1* (*NEAT1_1* + *NEAT1_2*) and *NEAT1_2* in starved cells compared to cells grown in full media, was analyzed by RT-qPCR. (E) Cells were treated as described in (D) and subjected to *NEAT1_2*- (green) and NONO- (red) specific co-immuno-FISH analyses. The nuclei are visualized by DAPI (blue). Scale bar is 10µm. (F) *NEAT1_2* signals from the experiment described in (E) were quantified in at least 200 cells per condition from three independent experiments. Statistical significance was determined using unpaired Student’s t-tests. ** , $p \leq 0.01$; ****, $p \leq 0.0001$

RNA-Seq

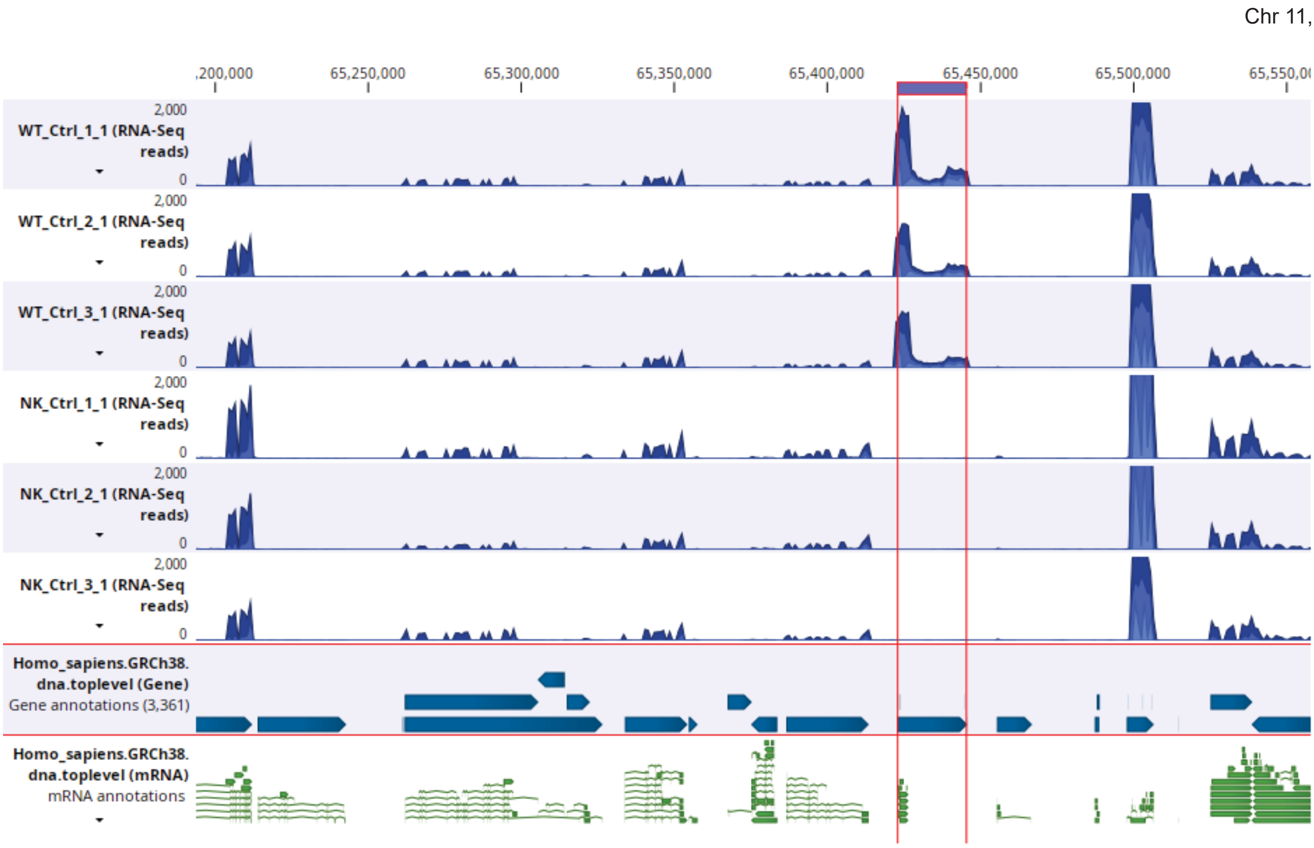

Fig. S2. RNA-seq analyses confirm deletion of the *NEAT1* genomic loci in the MCF7 *NEAT1* knockout cell line (MCF7 N1_KO). RNA-seq read tracks generated by CLC Genomic Workbench v22 encompassing the *NEAT1* genomic region at chromosome 11 (GRCh38, Ensembl annotation v104).

Journal of Cell Science • Supplementary information

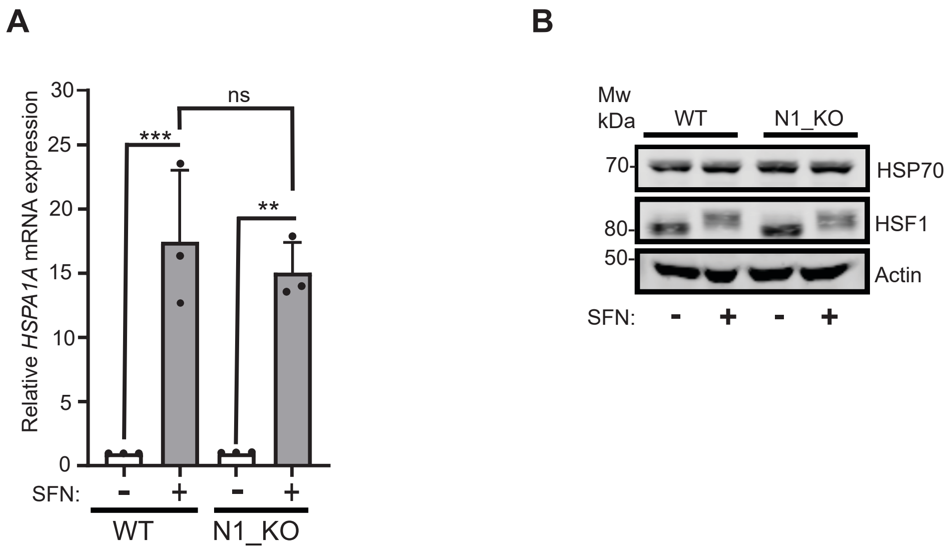

Fig. S3. SFN equally promotes the heat shock response in wild type and *NEAT1* knockout cells. (A) MCF7 wild type and *NEAT1* knockout cells (MCF7 N1_KO) were treated with SFN for 6 h. RNA was isolated and the relative expression of *HSPA1A* in SFN-treated cells versus control cells was determined in both cell lines by RT-qPCR. Figure is made from three independent experiments (n=3; **, p≤ 0.01; ***, p≤0.001; ns, not significant; unpaired Student's t-test). (B) HSF1 is activated by SFN in both wild type and *NEAT1* knockout cells. Immunoblot analyses of control and SFN-treated MCF7 wild type and N1_KO cells using an antibody towards HSF1. Activation of HSF1 is visualized as a mobility shift in SDS-PAGE.

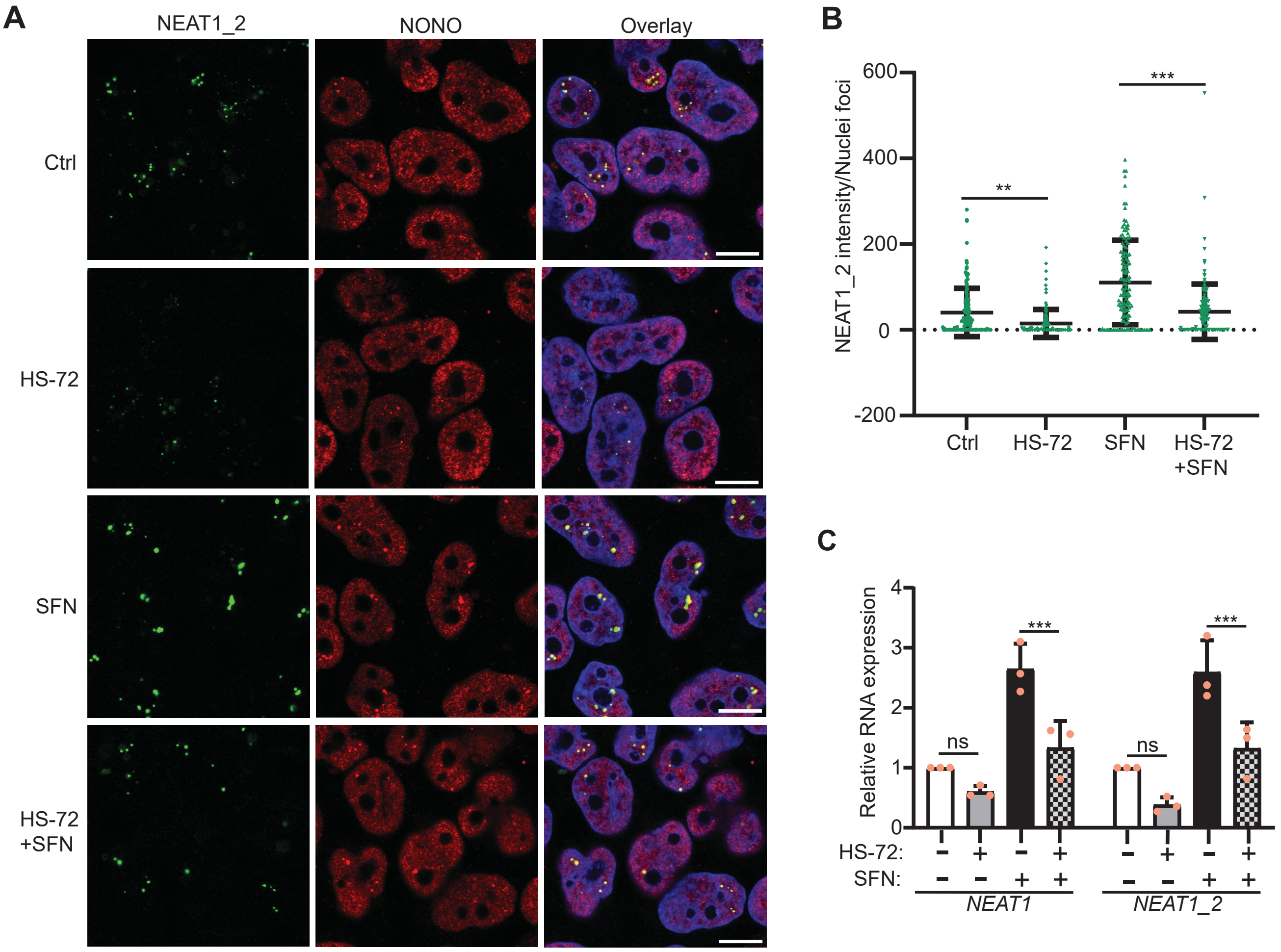

Fig. S4. The Hps70 inhibitor HS-72 reduces paraspeckle formation and *NEAT1* expression. HeLa Flp-In cells were left untreated or treated with 20 µM SFN for 6 h in the absence or presence of 25 mM HS-72. Paraspeckles were visualized by *NEAT1_2* (green channel)- and NONO (red channel)-specific co-immuno-FISH analyses. (B) The intensity of the *NEAT1_2* signals was quantified in at least 200 cells per cell line treated as described above, using the ImageJ software. The quantification was done across three independent experiments. Statistical analyses were performed with a one-way Anova with Tukey's multiple comparison test. **, p≤ 0.01; ***, p≤0.001. (C) HS-72 reduces SFN-induced *NEAT1* expression. Cells were treated as described in (A) and RNA was isolated. Relative expression of *NEAT1* (*NEAT1_1* + *NEAT1_2*) and *NEAT1_2* in treated versus untreated cells was determined by RT-qPCR analyses. Statistical analyses were performed using two-way Anova with a Tukey's multiple comparison test. *, p≤0.05; **, p≤ 0.01.

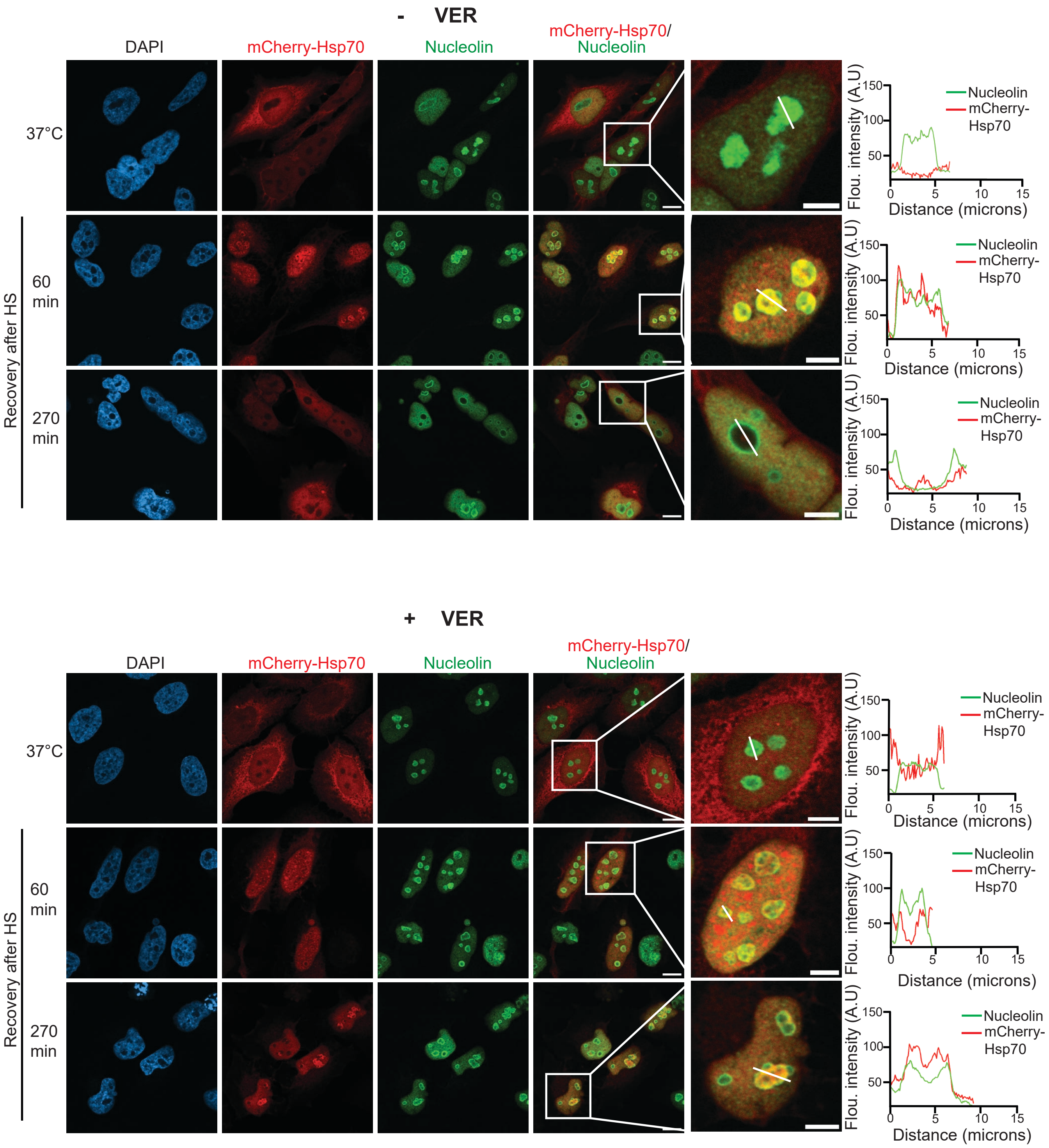

Fig. S5. Ectopic mCherry-Hsp70 co-localizes with nucleolin in nucleolar structures. HeLa Flp-In_GFP-SFPQ/mCherry-Hsp70 cells were left at 37°C or exposed to HS (43°C, 30 min) in the absence (upper panel) or presence (lower panel) of VER-155008 followed by recovery at 37°C for 60 or 270 min. Fixed cells were stained with an anti-nucleolin antibody and inspected by confocal microscopy. Co-localization of mCherry-Hsp70 (red channel) and nucleolin (green channel) is shown. DAPI visualizes the nuclei. Scale bar is 10 µm and 5 µm for the zoomed-in images.

Journal of Cell Science • Supplementary information

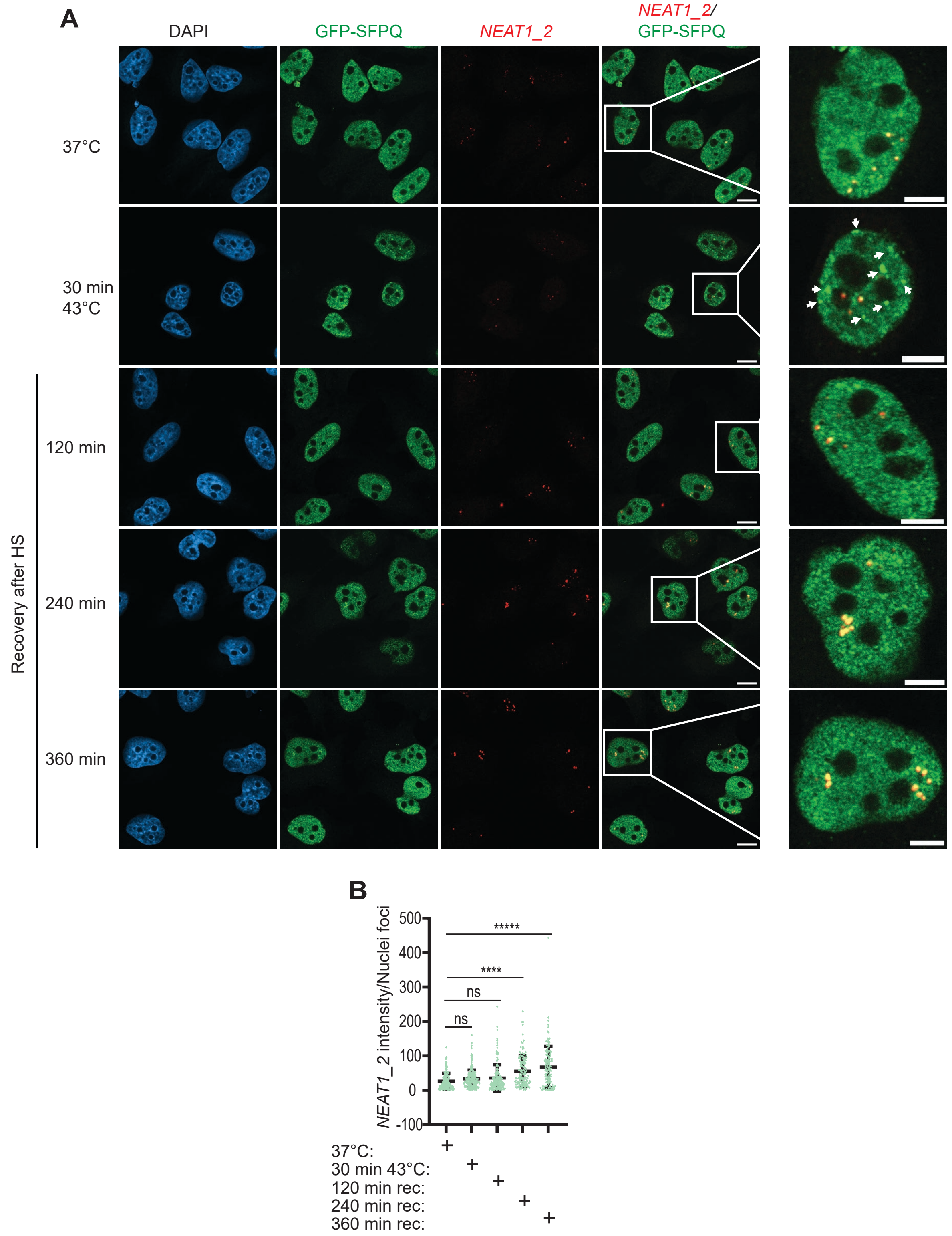
A
DAPI
GFP-SFPQ
NEAT1_2
NEAT1_2/
GFP-SFPQ
37°C
30 min
43°C
Recovery after HS
120 min
240 min
360 min
B
NEAT1_2 intensity/Nuclei foci
500
400
300
200
100
0
-100
*****
****
ns
ns
37°C:
30 min 43°C:
120 min rec:
240 min rec:
360 min rec:
+
+
+
+
+

Fig. S6. *NEAT1_2* co-localizes with GFP-SFPQ in paraspeckles during recovery from heat shock. HeLa Flp-In_GFP-SFPQ/mCherry-Hsp70 cells were left at 37°C or exposed to HS (43°C, 30 min) followed by recovery at 37°C for the indicated time points. Co-localization of GFP-SFPQ and *NEAT1_2* in paraspeckles is demonstrated by *NEAT1_2* (red)- and anti-GFP (green)-specific co-immuno-FISH analyses. The nuclei are visualized by DAPI (blue). The arrows indicate HS-induced GFP-SFPQ speckles with no colocalization with *NEAT1_2*. (B) *NEAT1_2* signals from the experiment described in (B) were quantified in at least 200 cells per condition from three independent experiments. Statistical analyses were performed with one-way Anova with Dunnett's multiple comparison test. ****, $p \leq 0.0001$; ns, not significant. Scale bar is 10 µm and 5 µm for the zoomed-in images.

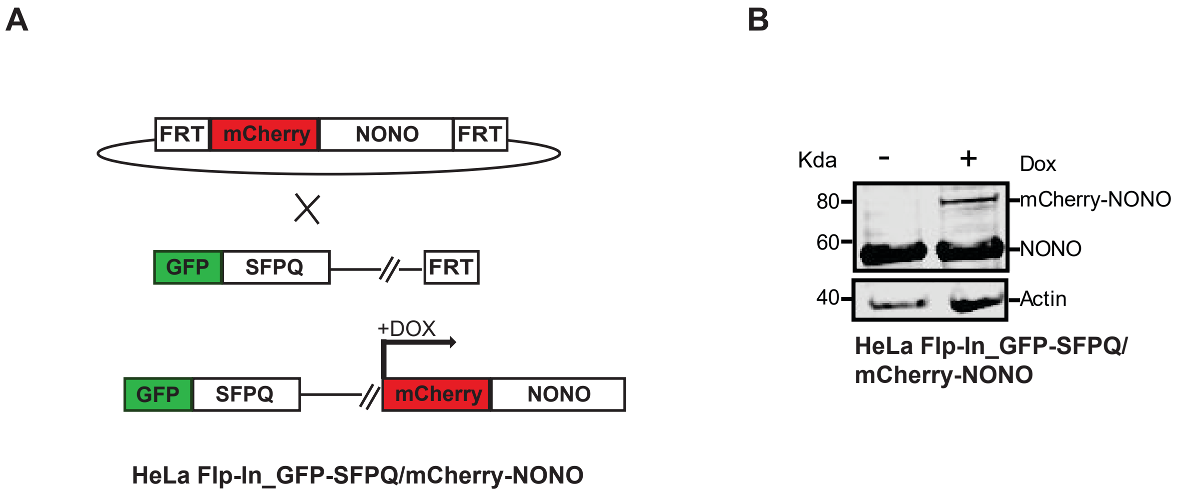

Fig. S7. Schematic illustration of the generation of the HeLa Flp-In_GFP-SFPQ/mCherry-NONO cell line. (A) A construct encoding mCherry-NONO was integrated into the FRT site of the HeLa Flp-In_GFP-SFPQ cell line, which allows doxocyline (DOX)-inducible expression of mCherry-NONO. (B) The HeLa Flp-In_GFP-SFPQ/mCherry-NONO cell line expresses mCherry-NONO in a DOX-inducible manner. mCherry-NONO and endogenous NONO are visualized by immunoblot analyses using an anti-Hsp70 antibody. Protein loading is visualized by re-probing the membrane with an anti-actin antibody.

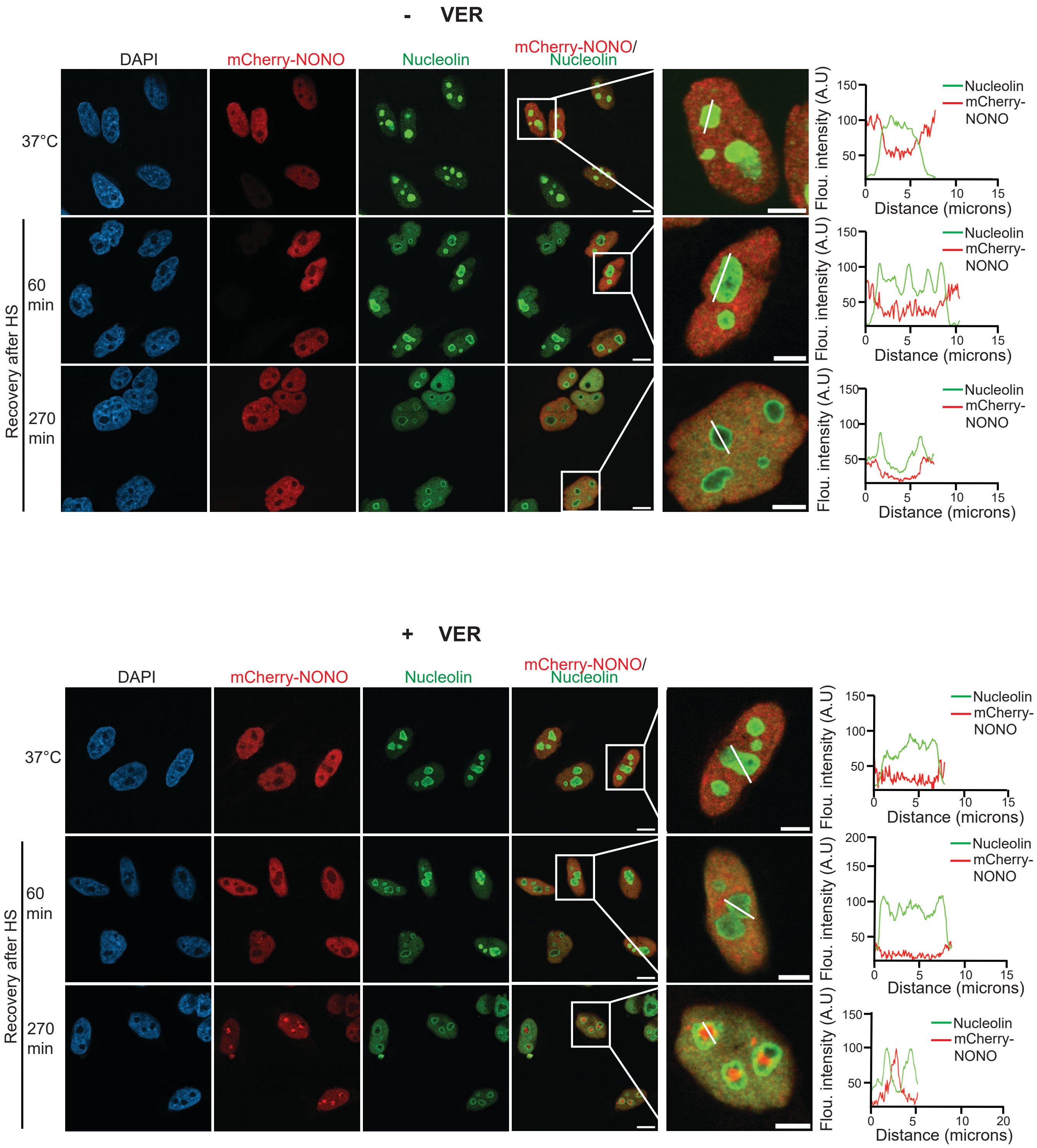

Fig. S8. Ectopic mCherry-NONO is retained in nucleoli structures upon inhibition of Hsp70 activity. HeLa Flp-In_GFP-SFPQ/mCherry-NONO cells were left at 37°C or exposed to HS (43°C, 30 min) in the absence (upper panel) or presence (lower panel) of VER-155008 followed by recovery at 37°C for 60 or 270 min. Fixed cells were stained with an anti-nucleolin antibody and inspected by confocal microscopy. Co-localization of mCherry-NONO (red channel) and nucleolin (green channel) upon VER-155008 treatment is shown. DAPI visualizes the nuclei. Scale bar is 10µm and 5µm for the zoomed-in images.

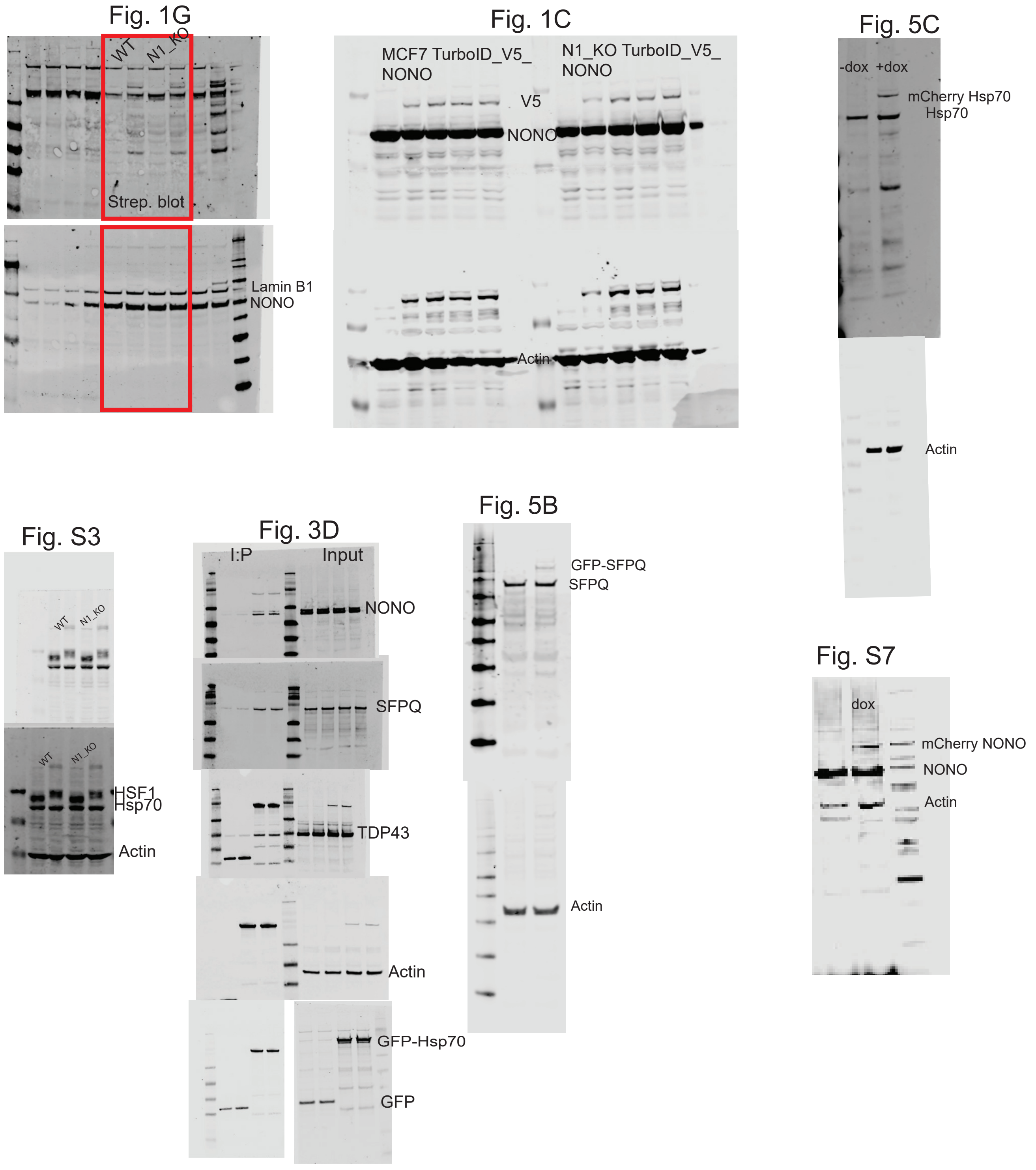

Fig. S9. Blot Transparency

Journal of Cell Science • Supplementary information

Table S1.

Available for download at
https://journals.biologists.com/jcs/article-lookup/doi/10.1242/jcs.264115#supplementary-data

Table S2.

Available for download at
https://journals.biologists.com/jcs/article-lookup/doi/10.1242/jcs.264115#supplementary-data

Table S3. Primer and guide RNA (sgRNA) sequences

Name	Primer sequence (5'-3')
**RT-qPCR**	
GAPDH	F: GAGCGAGATCCCTCCAAAAT
	R: AAATGAGCCCCAGCCTTCT
NEAT1	F: TCGGGTATGCTGTTGTGAAA
	R: TGACGTAACAGAATTAGTTCTTACCA
NEAT1_2	F: CGGAGGGTCTTGTAACACCAG
	R: AGTCCGGGCAACACAGAAAG
HSPA1A	F: GGGCCTTTCCAAGATTGCTG
	R: TGCAAACACAGGAAATTGAGAACT
**Cloning**	
attB_Hsp70	F: GGGGACAAGTTTGTACAAAAAAGCAGGCTCCACCGCGGCCAAAGCCGCGGCGATCGGC
	R: GGGGACCACTTTGTACAAGAAAGCTGGGTCTAATCTACCTCCTCAATGGTGGG
attB_NONO	F: GGGGACAAGTTTGTACAAAAAAGCAGGCTCTGCGCAGAGTAATAAAACTTTTAAC
	R: GGGGACCACTTTGTACAAGAAAGCTGGGTTTAGTATCGGCGACGTTTGTTTGG
**CRISPR sgRNA sequences**	
*NEAT1*_sgRNA_T1	TCCCTCCCTGTCGCTAACTC
*NEAT1*_sgRNA_T2	GCAAAACCTGAGTGCGGCCA

Journal of Cell Science • Supplementary information

Table S4. Antibodies used in this study

Name	Species	WB dilution	IF dilution	Company (Catalog #)
GFP	Rabbit	1:1000		ThermoFisher Scientific (A-11122)
NONO	Mouse	1:1000	1:200	BD Bioscience (611279)
V5	Mouse	1:1000	1:500	ThermoFisher Scientific (R960-25)
Hsp70	Rabbit	1:1000		Cell Signaling Technology (4872)
Hsp70	Rat	1:1000		Cell Signaling Technology (4873)
SFPQ	Mouse	1:1000	1:200	Santa Cruz (sc-101137)
PSPC1	Rabbit	1:1000	1:200	Sigma-Aldrich (SAB4200068)
TDP-43	Rabbit	1:1000		Proteintech (10782-2-AP)
Nucleolin	Mouse		1:200	ThermoFisher Scientific (39-6400)
Lamin-B1	Rabbit	1:5000		Proteintech (12987-1-AP)
Actin	Mouse	1:5000		Millipore (MAB1501)
800CW anti-rabbit	Goat	1:10000		LI-COR Bioscience (926-32211)
680LT anti-mouse	Goat	1:10000		LI-COR Bioscience (926-68020)
680LT anti-rat	Goat	1:10000		LI-COR Bioscience (926-68029)
680LT streptavidin		1:5000		LI-COR Bioscience (926-68031)

Journal of Cell Science • Supplementary information

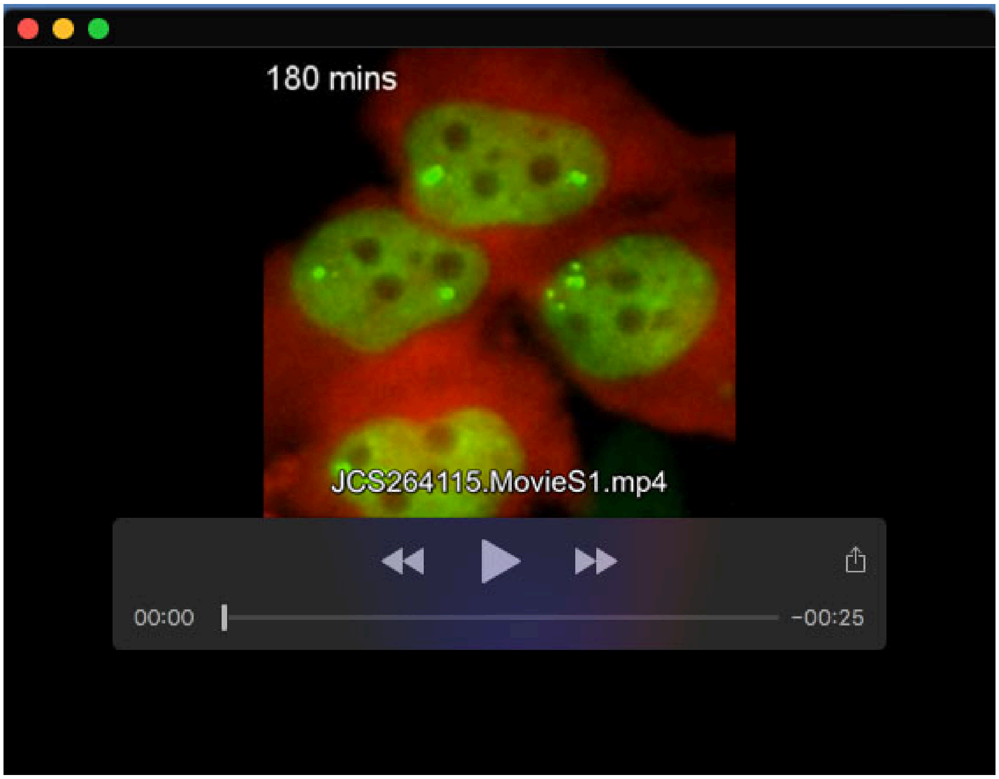

Movie 1. Time lapse analyses of HeLa Flp-In_GFP-SFPQ/mCherry-Hsp70 cells treated with 20 μM SFN.

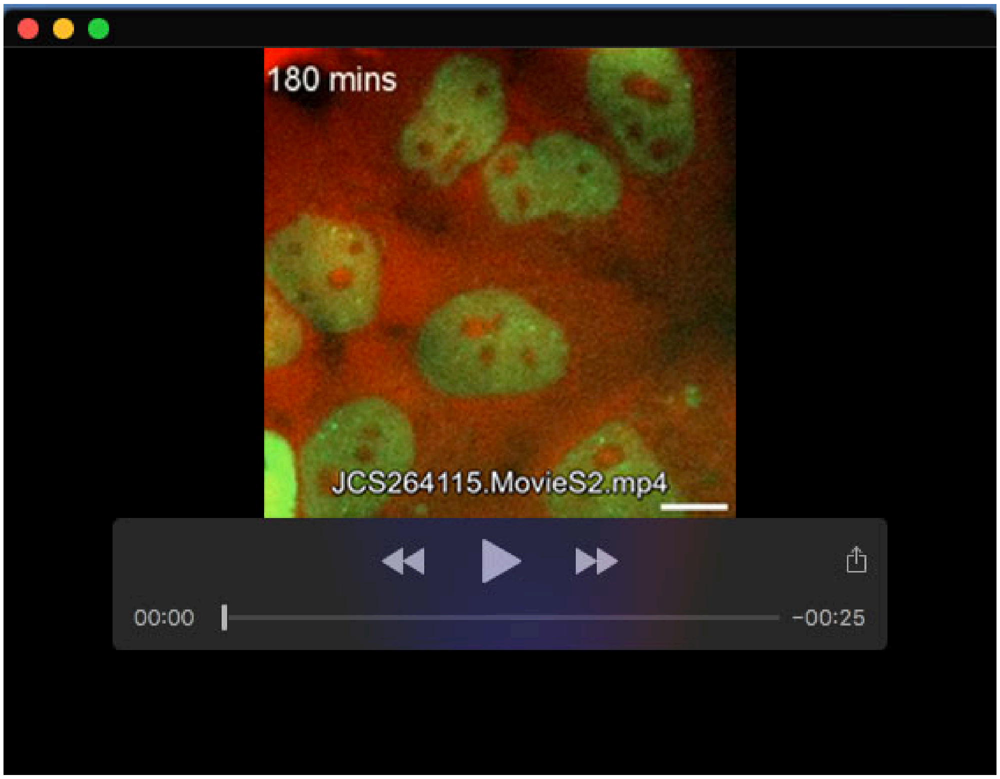

Movie 2. Time lapse analyses of HeLa Flp-In_GFP-SFPQ/mCherry-Hsp70 cells treated with 25 μM VER-155008 and 20 μM SFN.

Journal of Cell Science • Supplementary information

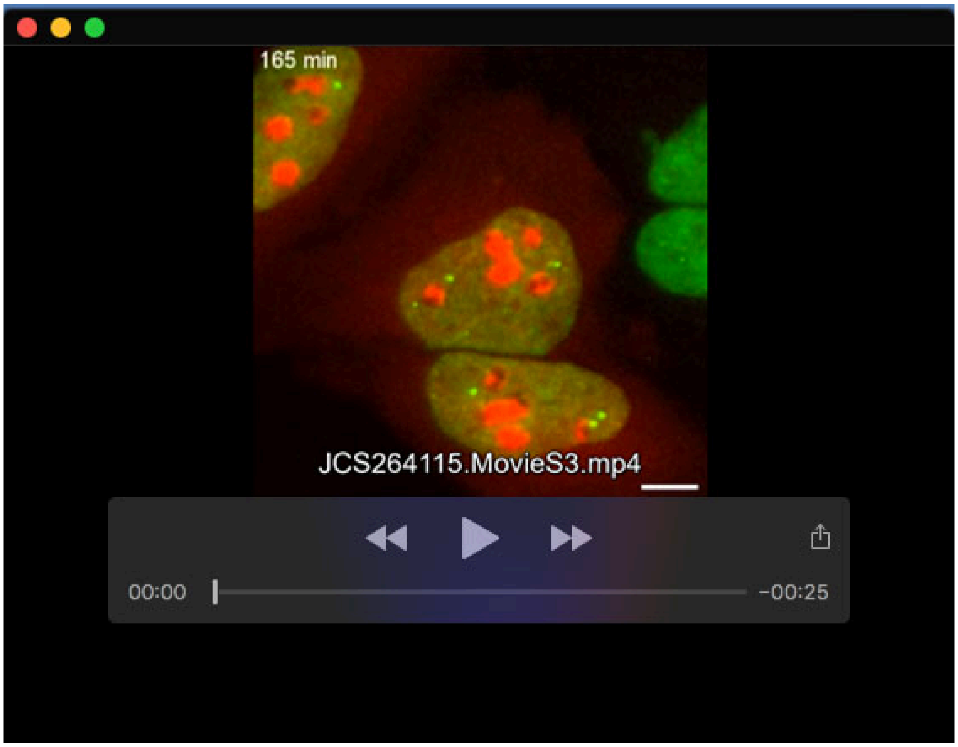

Movie 3. Time lapse analyses of HeLa Flp-In_GFP-SFPQ/mCherry-Hsp70 cells subjected to heat shock (43°C, 30 min) and left to recover at 37°C.

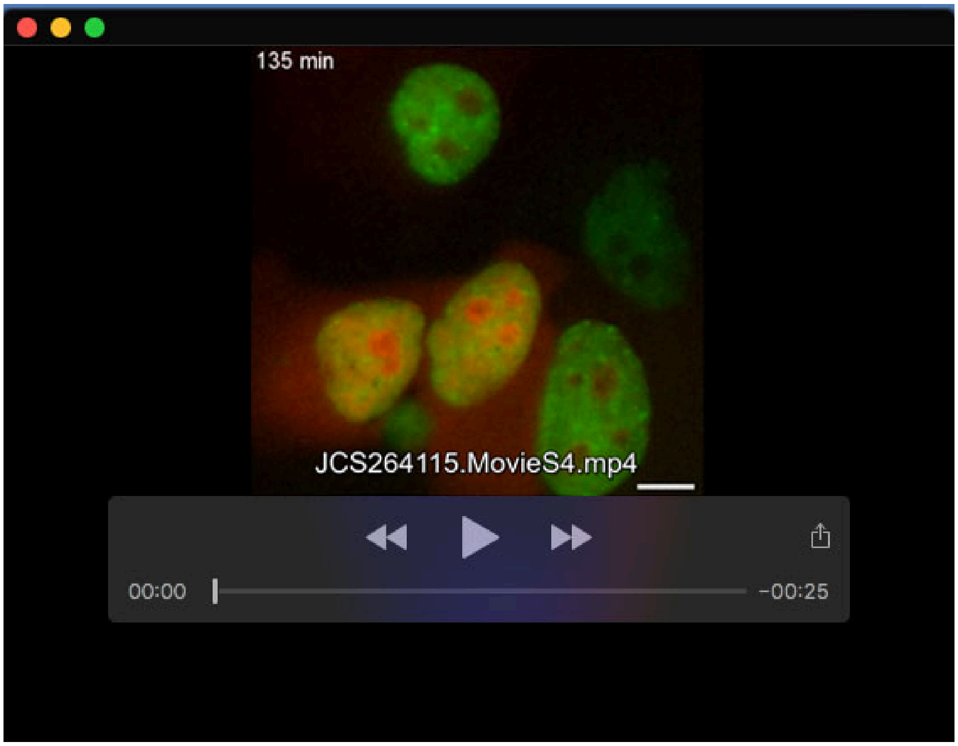

Movie 4. Time lapse analyses of HeLa Flp-In_GFP-SFPQ/mCherry-Hsp70 cells that were treated with 25 µM VER-155008 and subjected to heat shock (43°C, 30 min) and left to recover at 37°C.

Journal of Cell Science • Supplementary information

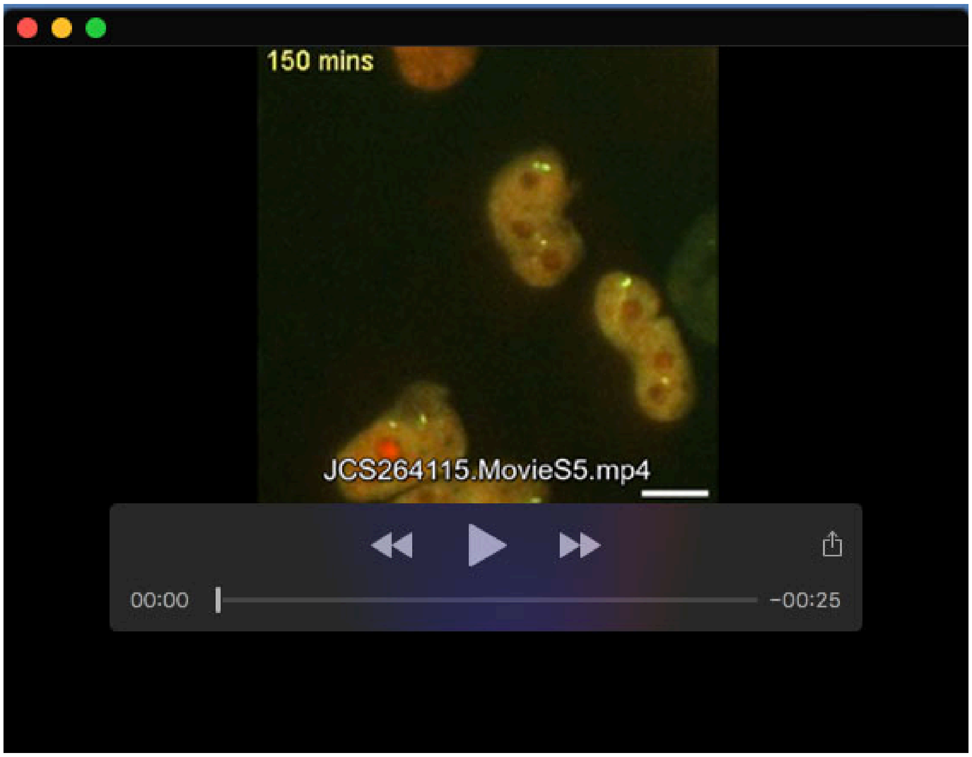

Movie 5. Time lapse analyses of HeLa Flp-In_GFP-SFPQ/mCherry-NONO cells subjected to heat shock (43°C, 30 min) and left to recover at 37°C.

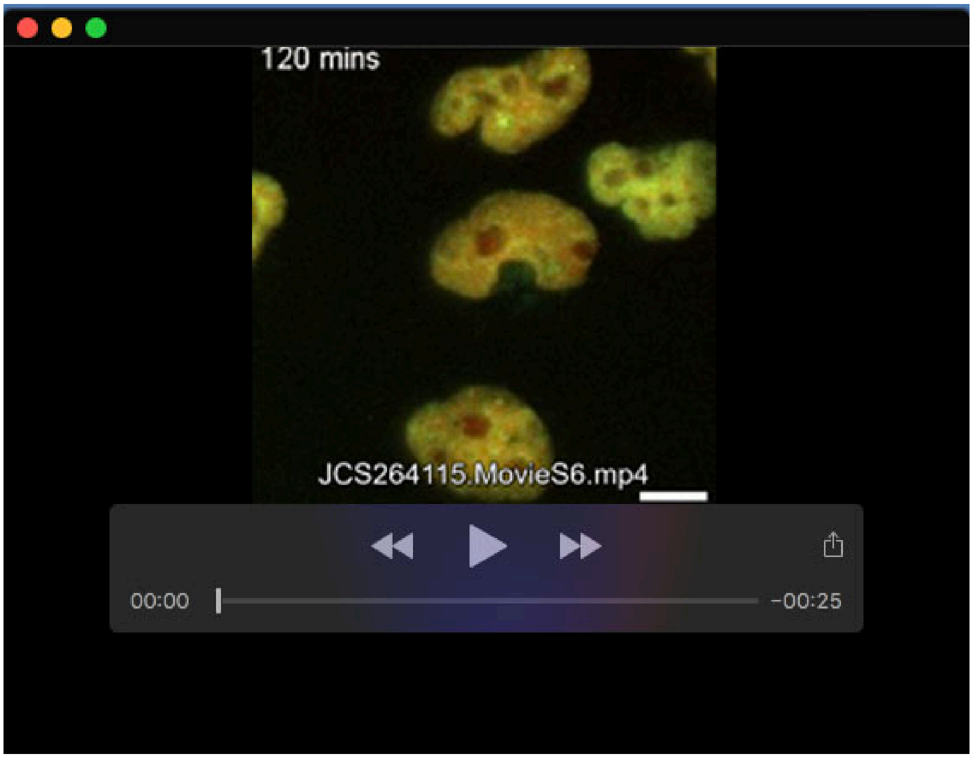

Movie 6. Time lapse analyses of HeLa Flp-In_GFP-SFPQ/mCherry-NONO cells that were treated with 25 µM VER-155008 and subjected to heat shock (43°C, 30 min) and left to recover at 37°C.

Journal of Cell Science • Supplementary information
